# Supplementary material for: Overview of Systematic Reviews of Health Interventions for the Prevention and Treatment of Overweight and Obesity in Children
Source: Nutrients. 2023 Feb 2;15(3):773. doi: 10.3390/nu15030773 (PMC9921486; doi:10.3390/nu15030773)
Supplement: Supplementary file 1 [file nutrients-15-00773-s001.zip › nutrients-2190307-supplementary.pdf]

Table S1. Detail search strategy per database

PubMed (MEDLINE)	((((obesity) AND (overweight)) AND (prevention)) AND (treatment)) AND (child) Filters: Systematic Review Sort by: Most Recent
EMBASE	Systematic review AND child AND obese and overweight AND prevention AND treatment - filter: Reviews
LILACS	obesity AND overweight AND prevention AND treatment AND child - Tipo de estudio: Rev Sistemática
CINAHL	obesity AND overweight AND treatment AND prevention AND ((TI (systematic* n3 review*)) or (AB (systematic* n3 review*)) or (TI (systematic* n3 bibliographic*)) or (AB (systematic* n3 bibliographic*)) or (TI (systematic* n3 literature)) or (AB (systematic* n3 literature)) or (TI (comprehensive* n3 literature)) or (AB (comprehensive* n3 literature)) or (TI (comprehensive* n3 bibliographic*)) or (AB (comprehensive* n3 bibliographic*)) or (TI (integrative n3 review)) or (AB (integrative n3 review)) or (JN "Cochrane Database of Systematic Reviews") or (TI (information n2 synthesis)) or (TI (data n2 synthesis)) or (AB (information n2 synthesis)) or (AB (data n2 synthesis)) or (TI (data n2 extract*)) or (AB (data n2 extract*)) or (TI (medline or pubmed or psyclit or cinahl or (psycinfo not "psycinfo database") or "web of science" or scopus or embase)) or (AB (medline or pubmed or psyclit or cinahl or (psycinfo not "psycinfo database") or "web of science" or scopus or embase)) or (MH "Systematic Review") or (MH "Meta Analysis") or (TI (meta-analy* or metaanaly*)) or (AB (meta-analy* or metaanaly*))). Narrow by SubjectAge: - all child
PsychINFO	(obesity and overweight and prevention and obesity and child and systematic review).mp. [mp=title, abstract, heading word, table of contents, key concepts, original title, tests & measures, meshlimit 2 to ("reviews (best balance of sensitivity and specificity)" and 180 school age <age 6 to 12 yrs> and "0110 peer-reviewed journal")
PROSPERO	child and obesity or overweight and treatment or prevention
TripDataBase	(title:child)(title:obesity OR overweight)(title:prevention OR treatment)
DARE	(child) AND (obesity OR overweight) AND (prevention OR treatment) IN DARE
OTSeeker	[Any Field] like 'child' AND [Any Field] like 'obesity' OR [Any Field] like 'overweight'
Epistemonikos	"(title:((title:(child) OR abstract:(child)) AND (title:(obesity) OR abstract:(obesity)) OR (title:(overweight) OR abstract:(overweight)) AND (title:(treatment) OR abstract:(treatment)) OR (title:(prevention) OR abstract:(prevention))) OR abstract:((title:(child) OR abstract:(child)) AND (title:(obesity) OR abstract:(obesity)) OR (title:(overweight) OR abstract:(overweight)) AND (title:(treatment) OR abstract:(treatment)) OR (title:(prevention) OR abstract:(prevention)))) (title:((title:(child) OR abstract:(child)) AND (title:(obesity) OR abstract:(obesity)) OR (title:(overweight) OR abstract:(overweight)) AND (title:(treatment) OR abstract:(treatment)) OR (title:(prevention) OR

	abstract:(prevention))) OR abstract:((title:(child) OR abstract:(child)) AND (title:(obesity) OR abstract:(obesity)) OR (title:(overweight) OR abstract:(overweight)) AND (title:(treatment) OR abstract:(treatment)) OR (title:(prevention) OR abstract:(prevention)))) Publication type: systematic reviews"
Health Evidence	"[obesity AND overweight AND prevention AND treatment] AND Limit:<br>Population = Grade school aged (5-12 years)<br>Topic Area = Chronic Diseases -> Obesity, Health Through the Ages -> Youth Health, Nutrition, Physical Activity, Social Determinants of Health (e.g., social environments, education, employment and working conditions)"
The Cochrane Library	(obesity):ti,ab,kw AND (overweight):ti,ab,kw AND (prevention):ti,ab,kw AND (treatment):ti,ab,kw AND ("Child"):ti,ab,kw

Table S2. Characteristic of the interventions and methods of each primary article.

Author & Year	Precondition			Stakeholder involvement			Approach				Content		Intervention			
	Policy & environment	Training	Included in curriculum	Community and parents	Educative personnel	Health Professionals	Peer education	Children participation	Parent participation	Educational intervention	Skilled based	Counselling	Nutritional/ Diet	PA/ Exercise	Behavioral change	Sedentary behaviors
PREVENTION & TREATMENT																
Alexandrow 1992						X		X	X	X		X	X		X	
Alvirde-García 2013				X			X	X			X	X	X	X	X	
Bacardí-Gascon 2012				X			X	X			X		X	X		
Baxter 2009								x								
Bush 1989	X	X	X		X		X	X	X	X	X				X	
Caballero 2003			X		X		X	X		X	X		X	X	X	
Chiodera 2008								X	X		X		X	X		
Chomitz 2010	X			X	X	X		X	X	X	X		X	X	X	
Colin-Ramírez 2009			X	X	X			X		X	X		X	X		
Datar and Sturm 2004			X					X		X	X			X		

Author & Year	Precondition			Stakeholder involvement			Approach				Content		Intervention			
	Policy & environment	Training	Included in curriculum	Community and parents	Educative personnel	Health Professionals	Peer education	Children participation	Parent participation	Educational intervention	Skilled based	Counselling	Nutritional/ Diet	PA/ Exercise	Behavioral change	Sedentary behaviors
Donnelly 2009			X		X			X		X	X			X		
Dzewaltowski 2010	X	X		X	X		X	X	X	X	X	X	X	X	X	X
Elizondo-Montemayor 2013				X	X			X			X		X	X		
Fernandes 2010	X		X		X			X		X	X			X		X
Fernandes and Strum 2011					X		X	X				X			X	
Foster 2008	X		X	X	X		X	X	X	X	X		X		X	
Fox 2009	X			X				X			X		X			
Gentile 2009	X			X	X			X	X	X	X		X	X	X	X
Graf 2005			X					X		X	X		X	X		
Greening 2011	X		X	X	X		X	X	X	X	X		X	X	X	X
Harrison 2011			X		X	X	X	X		X	X	X	X	X	X	X
Heelan 2009				X	X			X	X		X			X		
Henry 2006	X				X		X	X			X		X			
Hernández 2003	X				X			X		X	X		X			
Hinrichs 2010	X				X			X		X	X		X			
Hoelscher 2010				X	X			X		X	X		X	X		
Hollar 2010	X		X	X	X			X		X	X		X	X	X	
James 2004								X		X		X	X			
Jansen 2011 b	X		X	X	X			X		X	X	X			X	
Johnson 2012	X			X	X	X	X	X	X	X	X		X	X	X	X
Jones 2003	X							X			X		X			
Jordan 2008	X		X	X	X	X	X	X	X	X	X		X	X	X	
Kriemler 2010	X		X	X	X			X	X		X			X		
Levy 2012							X	X		X	X	X	X	X		
Luepker 1996		X	X	X	X		X	X	X	X	X		X	X	X	

Author & Year	Precondition			Stakeholder involvement			Approach				Content		Intervention			
	Policy & environment	Training	Included in curriculum	Community and parents	Educative personnel	Health Professionals	Peer education	Children participation	Parent participation	Educational intervention	Skilled based	Counselling	Nutritional/ Diet	PA/ Exercise	Behavioral change	Sedentary behaviors
Manios 2002			X		X		X	X	X	X	X		X	X		
Millimet 2008	X			X				X			X		X			
Millimet 2009	X			X				X			X		X			
Millimet 2010	X			X				X			X		X			
Muckelbauer 2009			X		X			X		X	X		X			
Ramírez-López 2005	X			X				X			X		X			
Robinson 1999		X	X		X		X	X		X	X				X	X
Safdie 2013							X	X		X	X		X	X		
Schieri 2009			X		X			X		X	X		X			
Siegrist in press	X		X	X	X		X	X		X	X		X	X	X	X
Veugelers 2005	X		X	X	X	X	X	X	X	X	X		X	X		X
Williamson 2012	X		X	X	X		X	X		X	X		X	X	X	
Zhu 2010	X		X	X	X		X	X	X	X	X	X	X	X	X	X
**TREATMENT**																
Alves 2008				X				X						X		
Aragona 1975				X		X		X	X	X	X	X	X	X	X	X
Arauz Bourdreau 2013				X		X		X	X	X	X	X	X	X	X	X
Barkin 2011				X				X	X		X	X		X	X	
Barthrellou 2010				X		X		X	X		X	X			X	
Berry 2007				X				X	X	X	X	X	X	X	X	
Berry 2014				X			X	X	X	X	X	X	X	X	X	
Boutelle 2011	X			X		X		X	X		X		X	X	X	X
Boutelle 2014						X		X			X	X	X		X	
Bryant 2011		X		X				X	X	X	X	X		X	X	X
Collins 2011				X		X		X	X	X	X	X	X		X	
Coppins 2011				X				X	X	X			X	X	X	

Author & Year	Precondition			Stakeholder involvement			Approach				Content		Intervention			
	Policy & environment	Training	Included in curriculum	Community and parents	Educative personnel	Health Professionals	Peer education	Children participation	Parent participation	Educational intervention	Skilled based	Counselling	Nutritional/ Diet	PA/ Exercise	Behavioral change	Sedentary behaviors
Creso 2012				X				X		X	X		X	X		
Croker 2011																
Croker 2012	X			X		X		X	X		X	X	X	X	X	
Davis 2013				X		X	X	X	X			X	X	X	X	
Davoli 2013				X		X			X	X		X	X	X	X	
De Niet 2012	X			X				X	X		X	X	X	X	X	
Díaz 2010			X	X	X	X		X	X	X	X	X	X		X	
Duffy 1993				X		X		X	X			X			X	
Duggins 2010	X			X		X	X	X	X	X	X	X	X	X		X
Eddy Ives 2012				X		X	X	X	X	X	X		X	X		
Epstein 1984a				X		X		X	X		X		X	X		
Epstein 1985a				X		X		X	X		X		X	X		X
Epstein 1985b				X		X		X			X		X	X	X	
Epstein 1985c				X		X		X	X	X	X	X			X	
Epstein 2000a				X		X		X	X	X	X	X			X	
Epstein 2001				X				X	X		X			X		X
Epstein 2005				X		X		X	X	X	X	X	X		X	
Epstein 2015		X		X		X		X	X		X	X	X			
Esfarjani 2013				X					X	X	X		X	X	X	X
Estabrooks 2009				X			X	X	X	X	X	X	X	X		X
Fraude 2010				X				X			X			X		
Flodmark 1993				X		X		X	X			X			X	
Gillis 2007				X		X		X		X	X		X	X		
Golan 2006				X		X		X	X	X	X	X	X	X	X	X
Golley 2007				X					X	X	X	X	X	X		X
Gunnarsdottir 2011a		X		X					X	X	X	X	X	X	X	

Author & Year	Precondition			Stakeholder involvement			Approach				Content		Intervention			
	Policy & environment	Training	Included in curriculum	Community and parents	Educative personnel	Health Professionals	Peer education	Children participation	Parent participation	Educational intervention	Skilled based	Counselling	Nutritional/ Diet	PA/ Exercise	Behavioral change	Sedentary behaviors
Hamilton-Shield 2014		X		X								X				
Ho 2016				X		X		X	X	X	X	X	X			
Hughes 2008				X		X		X				X	X		X	
Janicke 2008				X		X		X	X	X	X	X	X	X	X	X
Jansen 2011				X		X			X		X	X			X	
Kalarchian 2009				X		X		X	X	X		X			X	
Kalavainen 2007				X				X	X							
Kirk 2012				X		X		X		X	X		X	X		
Larsen 2015	X					X		X		X			X	X		
Lison 2012	X			X		X		X			X		X	X		
Lochrie 2013				X		X		X		X	X	X	X	X	X	
Looney 2014	X					X		X	X	X		X			X	
Maddison 2011	X							X			X			X		
Maddison 2014				X				X	X					X	X	X
Magarey 2011	X			X		X			X	X	X	X	X	X	X	X
Markert 2014	X			X		X			X			X	X	X	X	X
Mazzeo 2014				X		X			X	X	X	X	X	X	X	X
McCallum 2007								X	X				X	X	X	
Mirza 2013						X		X			X		X			
Moens 2012						X			X	X	X		X		X	
Munsch 2008				X		X		X	X			X	X	X	X	
NCT02436330	X			X				X		X		X	X	X	X	X
Nemet 2005						X		X			X		X	X	X	X
Nova 2001	X					X		X			X	X	X	X		
Nowicka 2009	X	X		X				X		X	X		X	X		
O'Connor 2013	X			X		X		X			X		X	X		X

Author & Year	Precondition			Stakeholder involvement			Approach				Content		Intervention			
	Policy & environment	Training	Included in curriculum	Community and parents	Educative personnel	Health Professionals	Peer education	Children participation	Parent participation	Educational intervention	Skilled based	Counselling	Nutritional/ Diet	PA/ Exercise	Behavioral change	Sedentary behaviors
Raynor 2012a				X		X			X		X	X	X		X	
Raynor 2012b				X		X			X		X	X	X	X	X	X
Reinehr 2010				X				X			X		X	X	X	X
Rensnicow 2015				X		X			X		X	X	X	X	X	
Resnick 2009									X					X		
Rodearmel 2007				X				X					X	X		
Sacher 2010				X			X	X		X	X		X	X	X	
Saelens 2013				X		X		X			X	X			X	
Satoh 2007									X				X			
Schwingshandl 1999								X	X				X	X		
Serra-Paya 2015	X							X			X	X	X	X	X	
Siwik 2013				X		X		X				X			X	
Small 2013				X		X			X	X		X	X	X		X
Taveras 2015								X	X				X	X	X	
Taylor 2015				X		X		X	X		X	X	X	X	X	
Van Grieken 2013		X				X			X			X	X	X	X	X
Vann 2013								X					X	X		
Wafa 2011								X			X			X		X
Wake 2009				X		X			X			X			X	
Wake 2013				X		X		X	X	X	X	X			X	
Waling 2010				X				X	X			X	X	X		X
Waling 2012				X				X	X			X	X	X	X	X
Warschburger 2016								X	X				X	X	X	
Weigel 2008						X		X			X		X	X	X	X*
Weitraub 2008				X				X			X			X		
West 2010				X		X	X	X	X		X	X	X	X	X	X

Author & Year	Precondition			Stakeholder involvement			Approach				Content		Intervention			
	Policy & environment	Training	Included in curriculum	Community and parents	Educative personnel	Health Professionals	Peer education	Children participation	Parent participation	Educational intervention	Skilled based	Counselling	Nutritional/ Diet	PA/ Exercise	Behavioral change	Sedentary behaviors
Wilfley 2007				X		X		X	X	X	X	X	X	X	X	
Woo 2004			X	X		X		X			X		X	X		
Whright 2012				X	X			X		X	X		X			

Supplementary table S3. Outcomes for diet or nutritional interventions.

Author & Year	Intervention	Outcome	Results Mean Difference IV [95% CI] ([a] Hedges'g)	Conclusion of the author	Author & Year of SR
**Prevention & Treatment**					
Baxter et, al. 2009	School breakfast program consumption	Mean change % breakfast in classroom compared to cafeteria	2.64[a]	Positive relationship between BMI and observed energy intake at school meals, and between BMI and school breakfast in the classroom;	Williams et, al. 2013
Foster et, al. 2008	School nutrition policy initiative	Adjusted change in BMI-SDS	-0.01 [-0.08, 0.06] [a]	Multicomponent school-based intervention can be effective in curbing the development of overweight among children in grades 4 through 6.	Williams et, al. 2013 Sbruzzi et, al. 2013
		Adjusted OR overweight	0.65 [0.54, 0.79] [a]*		
		Adjusted OR obesity	1.09 [0.85, 1.40] [a]		

		Dietary intake	↓ Calorie intake		
Fox et, al. 2009	À la carte LNED food not available	Adjusted change in BMI-SDS	-0.15 [-0.37,0.07] [a]	Policies that, potentially controlling children's BMI and in improving children's over- all diet quality may want to consider include limiting french fries and desserts in school lunches and limiting children's access to low-nutrient, energy-dense foods through vending machines.	Williams et, al. 2013
		Adjusted odds ratio obesity	1.09 [0.57,2.08] [a]		
	Milk not available for school lunch	Adjusted change in BMI-SDS	-0.13 [-0.33,0.07] [a]		
		Adjusted odds ratio obesity	1.17 [0.75,1.82] [a]		
	Fresh fruit/ raw vegetables available	Adjusted change in BMI-SDS	0.19 [0.01,0.37] [a]		
		Adjusted odds ratio obesity	1.13 [0.73,1.75] [a]		
	Fried potato products not available	Adjusted change in BMI-SDS	0.20 [<0.01,0.40] [a]		
		Adjusted odds ratio obesity	2.70 [1.58,4.62] [a]		
	Desserts offered ≤once a week	Adjusted change in BMI-SDS	0.08 [-0.08,0.24] [a]		
		Adjusted odds ratio obesity	1.78 [1.13, 2.80] [a]*		
Henry 2006	National school lunch program	Hedges' g overweight	1.39 [0.55, 2.24)] [a]*	-	Williams et, al. 2013
Hernández et, al. 2003	National school lunch program	Adjusted change in BMI (kindergarten)	0.12 [-0.33, 0.57]	Results suggest participation in the NSLP is associated with rapid weight gain for low-income girls but not for low-income boys.	Williams et, al. 2013
		Adjusted change in BMI (1st grade)	.20 [-0.29, 0.69]		
		Adjusted change in BMI (3rd grade)	0.36 [-0.25, 0.97]		
		Adjusted change in BMI (5th grade)	0.52 [-0.24, 1.28]		
Hinrichs 2010	National school lunch program	Adjusted change in BMI (male)	-0.02 (-0.06, 0.02)	The NSLP appears to have had no long-term effect on health but may have affected educational attainment.	Williams et, al. 2003
		Adjusted change in BMI (female)	-0.02 (-0.07, 0.03)		

		Change in prevalence of overweight (male)	<-0.01 (-0.01, <0.01) [a]		
		Change in prevalence of overweight (female)	<-0.01 [-0.01, <0.01] [a]		
		Change in prevalence of obesity (male)	<-0.01 [<-0.01, <0.01] [a]		
		Change in prevalence of obesity (female)	<-0.01 [<-0.01, <0.01] [a]		
James et, al. 2004	Discouraging consume of carbonated drinks vs. No intervention	BMI (kg/m^2)	-0.50 [-0.94, -0.06] *	A school based education programme produced a modest reduction in the number of carbonated drinks consumed, which was associated with a reduction in the number of overweight and obese children.	Sbruzzi et, al. 2013
		BMI z-score	-0.19 [-0.36, -0.02] *		
		Drinks consumed	↓ Carbonated drinks		
			Adjusted OR overweight and obesity:		
Jones et, al. 2003	National school lunch program	Food secure (male)	1.06 [0.53,2.08] [a]	Food assistance programs play a protective role for low income children's health, particularly in girls in food insecure households.	Williams et, al. 2003
		Food secure (female)	0.49 [0.22,1.10] [a]		
		Food insecure (male)	0.62 [0.25,1.54] [a]		
		Food insecure (female)	0.29 [0.11,0.80] [a]*		
	School breakfast and national school lunch programs	Food secure (male)	1.33 [0.81,2.18] [a]		
		Food secure (female)	0.66 [0.35,1.26] [a]		
		Food insecure (male)	0.85 [0.42,1.74] [a]		
		Food insecure (female)	0.42 [0.19,0.96] [a]*		
Millimet et, al. 2008 y 2010	National school lunch program	Change in probability of being overweight	0.13 [0.07, 0.20] [a]	The SBP is a valuable tool in the current battle against childhood obesity, whereas the NSLP exacerbates the current epidemic.	Williams et, al. 2003
		Change in probability of being obese	0.13 [0.05, 0.20] [a]		
	School breakfast program	Change in probability of being overweight	-0.07 [-0.14, <-0.01] [a]		
		Change in probability of being obese	-0.05 [-0.13, 0.03] [a]		
Millimet et, al. 2009	School breakfast program	Change in BMI growth rate 3rd grade	-0.03 [-0.06, <-0.01]	Positive and statistically significant association between School Breakfast program and child weight when using estimators that require conditional independence.	Williams et, al. 2003
		Change in probability of overweight 3rd grade	-0.21 (-0.33, -0.03)		
		Change in probability of obese 3rd grade	-0.17 (-0.26, -0.01)		

		Change in BMI growth rate 5th grade	-0.04 (-0.08, 0.01)		
		Change in probability of overweight 5th grade	-0.28 (-0.40, -0.09)		
		Change in probability of obesity 5th grade	-0.12 (-0.28, -0.04)		
Muckelbaurer et, al. 2009	Environmental and education on water consumption on the incidence of overweight vs. No intervention	BMI (kg/m²)<br>BMI z-score<br>Self reported water consumption	-0.30 [-0.53, -0.07] *<br>-0.08 [-0.16, -0.00]<br>↑ Water intake	A school-based intervention solely focusing on the promotion of water consumption was effective for decreasing the incidence of overweight.	Sbruzzi et, al. 2013
Sichieri et, al. 2009	Education program to discourage sugar-sweetened beverages consumption	BMI (kg/m²)<br>24h recalls beverage consumption	0.20 [-0.24, 0.64]<br>↓ Carbonated drinks	The single message of cutting down on sodas was effective only among over- weight girls, suggesting that efforts to reduce energy intake though liquids should also emphasise overall sweetened beverages, including the addition of sugar to juices.	Sbruzzi et, al. 2013
Ramirez-Lopez et, al. 2005	School breakfast program	Change in BMI<br>Change in BF%<br>Change in prevalence of overweight or obesity<br>Change prevalence of obesity	NR<br>NR<br>NR<br>NR	Study results showed no evidence of a negative effect of SBP in terms of risk factors for obesity and cardiovascular disease.	Williams et, al. 2013
Wright et, al. 2012	Nutrition education	Intake of vegetables<br>Intake of 100% fruit juices<br>Intake of fruits<br>Self-efficacy<br>â BMI (kg/m2	↑ Intake of vegetables<br>↑ Intake of 100% fruit juices<br>↑ Intake of fruits<br>NR<br>NR	This study supports the effectiveness of a culturally sensitive, family-centered CSHP to improve dietary behaviors and to decrease BMI z-scores in a sample of predominately Mexican-American, low-income, school- aged children.	Williams et, al. 2013<br>Mead et, al. 2017
**Treatment**					
Ho et, al. 2016		Change in BMI (kg/m²)	-0.12 [-0.85, 0.61]		

	Use of portion control too and nutrition counselling vs. nutrition counselling alone	BMI z-score	-0.5 [-0.17, 0.07]	Addition of a portion control tool to standard nutritional counseling did not result in a significant change in BMI z score after 6 months. Nutritional counseling for families did result in improvement in BMI z score over a 6-month period, but there remains a need for simple, adjunctive, practical tools that can assist families in weight management when they have children who are overweight and obese.	Mead et, al. 2016
		Weight (kg)	-0.2 [-2.45,2.21]		

Supplementary table S4. Outcomes for physical activity or exercise interventions.

Author & Year	Intervention	Outcome	Results Mean Difference IV [95% CI] ([a] Hedges'g)	Conclusion of the author	Author & Year of SR
**Prevention & Treatment**					
Chiodera et, al. 2008	Professionally led PE	Change in BMI grade 1	-0.21 [a*]	Professionally guided programs of physical education in the primary school lead to significant progresses in the development of conditional and	Williams et, al. 2013
		Change in BMI grade 2	-0.05 [a]		
		Change in BMI grade 3	-0.06 [a]		
		Change in BMI grade 4	0.04 [a]		
		Change in BMI grade 5	0.02 [a]		

				coordinative abilities, without altering BMI values.	
Datar et, al. 2004	Increased duration of PE (1 hr. per week)	Adjusted change in BMI, normal weight (male)	0.04 [-0.04,0.12] [a]	Expanding physical education programs in schools, in the form in which they currently exist, may be an effective intervention for combating obesity in the early years, especially among girls.	Williams et, al. 2013
		Adjusted change in BMI, normal weight (female)	0.01 [-0.07,0.10] [a]		
		Adjusted change in BMI, overweight or obese (male)	-0.07 [-0.19,0.05] [a]		
		Adjusted change in BMI, overweight or obese (female)	-0.32 [-0.46, -0.17] [a*]		
Donnelly et, al. 2009	PA in curriculum	BMI Hedges' g	0.01 [-0.09,0.11] [a]	Greater levels of exposure to PAAC lessons were associated with smaller increases in BMI and favorable shifts in BMI percentile.	Williams et, al. 2013
Fernandes 2010 & Fernandes et, al. 2011	National Association of Sport and Physical Education (NASPE) guidelines	PE duration Adjusted change in BMI %	-0.74 [-1.78, 0.30] [a]	Meeting the recommended levels of recess is associated with a lower BMI percentile for children between 1st and 5th grade while PE is effective only for boys.	Williams et, al. 2013
		PE duration Adjusted change in BMI (male)	-1.56 [-3.03, -0.09] [a*]		
		PE duration Adjusted change in BMI (female)	0.05 [-1.40,1.50] [a]		
		Break period duration: adjusted change in BMI %	-0.74 [-1.33, -0.15] [a*]		
		Break period duration: adjusted change in BMI % (male)	-0.81 [-1.67, 0.05] [a]		
		Break period duration: adjusted change in BMI % (female)	-0.69 [-1.49, 0.11] [a]		
Kriemler et, al. 2010	Multi-component physical activity program (structuring and adding PA sessions, daily short activity breaks, PA homework) vs. No intervention	Waist circumference	-0.50 [-1.78, 0.78]	A school based, multi-component physical activity intervention including compulsory elements improved physical activity and fitness and reduced adiposity in children	Sbruzzi et, al. 2013
		BMI (kg/m^2)	0.00 [-0.50, 0.50]		
		Systolic BP (mmHg)	-1.00 [-2.60, 0.60]		
		Diastolic BP (mmHg	-1.00 [-2.35, 0.35]		

Heelan et, al. 2009	Walking school bus scheme	Intervention vs. Control BMI-SDS Hedges' g	-0.21 [-0.58,0.15] [a]	Walking bus school increases the percentage of children who walk to and from school, as well as the frequency of walking.	Williams et, al. 2013 -
		Frequent v. passive BMI-SDS Hedges' g	-0.49 [-0.94, -0.03] [a*]		
		Infrequent v. passive BMI-SDS Hedges' g	-0.17 [-0.61,0.28] [a]		
		Intervention vs. Control BF% Cohen's d	-0.25 [-0.61,0.11] [a]		
		Frequent v. passive BF% Cohen's d	-0.59 [-1.05, -0.13] [a*]		
		Infrequent v. passive BF% Cohen's d	-0.28 [-0.72,0.17] [a]		
**Treatment**					
Alves et, al. 2008	Exercise vs. No-care	Change in BMI (kg/m²)	-0.53 [-1.05, -0.01] *	An exercise program for children, sustained for six months, was effective for reducing weight gain in overweight children living in a very poor neighborhood.	Mead et, al. 2017
		Change in Weight (kg)	-1.37 [-1.99, -0.75] *		
Faude et, al. 2010	Football training program vs. Standard sports program	Change in BMI (kg/m²)	-0.10 [-3.02, 2.82]	Football training is at least as efficacious in improving the physical capacity, health-related fitness parameters and self-esteem of overweight children as a standard exercise program	Mead et, al. 2017
		Change in BMI z-score	0.10 [-0.39, 0.59]		
		Change in Weight (kg)	0.30 [-10.26, 10.86]		
Maddison et, al. 2011	Active video game use vs. No-care	Change in BMI (kg/m²)	-0.25 [-0.47, -0.03] *	A home-based, family-delivered intervention to reduce all leisure-time screen use had no significant effect on screen-time or on BMI in	Mead et, al. 2017
		Change in BMI z-score	-0.06 [-0.30, 0.18]		
		Change in Weight (kg))	-0.72 [-3.76, 2.32]		

				overweight and obese children aged 9–12 years.	
Weintraub et, al. 2008	After-school team sports program vs. "Active placebo"	Change in BMI (kg/m²)	-0.57 [-4.94, 3.80]	An after-school team soccer program for overweight children can be a feasible, acceptable, and efficacious intervention for weight control.	Mead et, al. 2017
		Change in BMI z-score	-0.09 [-0.45, 0.27]		
					Podnar et, al. 2020

SD: Standard Deviation; CI: Confidence Interval; BMI: Body Mass Index; OR: Odds Ratio; NR: No Report; PA: physical activity; PE: physical education; BP: Blood pressure

Supplementary table S5. Outcomes for behavior change interventions.

Author & Year	Intervention	Outcome	Results<br>Mean Difference<br>IV [95% CI]<br>([a] Hedges'g)	Conclusion of the author	Author & Year of SR
For Prevention					
Alexandrov et, al. 1992	Parent-children counseling, children	BMI (kg/m²)<br>Systolic BP (mmHg)	-0.40 [-0.75, -0.05] *<br>0.10 [-1.37, 1.57]	The marked prevalence of risk factors among children	Sbruzzi et, al. 2013

	received individual sessions vs. Children group counselling	Diastolic BP (mmHg)	3.30 [1.78, 4.82] *	and teenagers and their high reproducibility in repeated studies indicate the need for corrective measures.	
Bush et, al. 1989	KYB curriculum vs. Usual curriculim	Systolic BP (mmHg)	-0.40 [-1.97, 1.17]	KYB may reduce chronic disease risk in diverse school populations, and that increased efforts should be made to improve implementation methods	Sbruzzi et, ,al. 2013
		Diastolic BP (mmHg	-2.80 [-4.10, -1.50] *		
For Treatment					
Boutelle et, al. 2014	Regulation of cues program vs. Usual care	Change in BMI (kg/m^2)	-0.1 (4.7292)	The ROC intervention may be useful with overweight and obese children. Larger, fully powered studies are needed to further evaluate the efficacy of this model.	Mead et, al. 2017
		Change in BMI z-score	-0.1 (0.4262)		
Jansen et, al. 2011	Parental cognitive behavioral treatment (CBT) vs. Waiting-list control	Change in BMI percentile (post intervention)	-2.3 (5.7)	The parents' treatment had significant effects on child and parent BMI.	Loveman et, al. 2015
		Change in BMI percentile (on longest follow-up)	-2.1 (5.7)		

[1] BMI: Body Mass Index; BP: Blood Pressure; mmHg: Mercury millimeters; CBT: Cognitive behavioral treatment

Supplementary Table S6. Outcomes in multi-component interventions.

**Author & Year**	**Intervention**	**Outcome**	**Results Mean Difference IV [95% CI] ([a] Hedges'g)**	**Conclusion of the author**	**Author & Year of SR**
**Prevention & Treatment**					

Author & Year	Intervention	Outcome	Results Mean Difference IV [95% CI] ([a] Hedges'g)	Conclusion of the author	Author & Year of SR
Alvirde-García et, al. 2013	Nutrition and physical education	Decreased total kcals<br>↓ bread consumption<br>↓ fat consumption<br>↓ sugar consumption<br>↓ Risk of obesity/ BMI (kg/m^2)	NR<br>NR<br>NR<br>NR<br>NR	School programs are helpful in tackling childhood obesity, but their benefits are not immediate.	Andrade et, al. 2018
Bacardí-Gascon et, al. 2012	Nutrition education and physical activity	↑ Vegetable intake<br>↓ fat and salt consumption	NR<br>NR	The results of this study indicate that with a comprehensive intervention there are positive responses in lifestyle changes and a moderate reduction in abdominal obesity	Andrade et, al. 2018
Caballero et, al. 2003	Multi-component school-based intervention (dietary intake, PA, classroom curriculum, family involvement) vs. No intervention	BMI (kg/m^2)	-0.20 [-0.66, 0.26]	Significant reductions in the fat content of school menus and in the dietary fat intake of children can be achieved by training and supporting food service staff.	Sbruzzi et, al. 2013
Chomitz et, al. 2010	Healthy Living Cambridge Kids	Change in BMI-SDS<br>Change in prevalence of overweight (points)<br>Change in prevalence of obesity (points)	-0.04 [a]*<br>0.6% [a]<br>-2.2% [a]	Our positive results add to the short, but growing list of studies showing that "upstream" oriented, multidimensional interventions with children, schools, and communities can curb and potentially prevent obesity.	Williams et. Al, 2013
Colín-Ramírez et, al. 2009	Nutrition education and physical activity	↓ BMI (kg/m^2)<br>↓ Sodium intake	NR<br>NR	The intervention managed to favorably modify the diet, physical activity and blood pressure of the children studied.	Andrade et, al. 2018
Crespo et, al. 2012	Nutrition education and physical activity	Family fruit and vegetable consumption<br>Change BMI (kg/m^2)	NR<br>NR	A promotora-based behavioral intervention was efficacious at changing parental factors and child obesity-related health behaviors.	Andrade et, al. 2018

Author & Year	Intervention	Outcome	Results Mean Difference IV [95% CI] ([a] Hedges'g)	Conclusion of the author	Author & Year of SR
Dzewaltowski et, al. 2010	Multi-component school-based intervention (HOP'N intervention: build healthy after-school environments) vs. No appliance of CATCH guidelines	BMI (kg/m^2)<br>BMI z-score	0.00 [-1.04, 1.04]<br>0.02 [-0.24, 0.28]	The HOP'N program had a positive impact on overweight/obese children's PA and after-school active recreation time.	Sbruzzi et,al. 2013
Elizondo-Montemayor et, al. 2013	Nutrition and physical education	BMI (kg/m^2)<br>↓ BP in intervention group<br>↓ Calorie intake	NR<br>NR<br>NR	A school-setting lifestyle intervention led to a decreased prevalence of being overweight/obese and to a striking reduction in the prevalence of the metabolic syndrome in a sample of Mexican children.	Andrade et, al. 2018
Gentile et, al. 2009	Decrease screen time, fruit and vegetable consume, PA levels at family, school and community vs. No intervention	BMI (kg/m^2)	0.00 [-0.07, 0.07]	Switch program yielded small-to-modest treatment effects for promoting children's fruit and vegetable consumption and minimizing screen time. The Switch program offers promise for use in youth obesity prevention.	Sbruzzi et,al. 2013
Graf et, al. 2005	Multi-component school-based intervention (CHILT project: health education and PA)	BMI (kg/m^2)	0.20 [-0.28, 0.68]	Preventive intervention in primary schools offers an effective means to improve motor skills in childhood and to break through the vicious circle of physical inactivity – motor deficits – frustration – increasing inactivity possibly combined with an excess energy intake and weight gain.	Sbruzzi et, al. 2013
Greening et, al. 2011	Promotion of healthy dietary habits and PA vs. One nutritional information session + weekly PA class + health education	Waist circumference	-3.40 [-4.86, -1.94] *	A population-based approach is recommended over a targeted approach to cultivate a culture of healthy lifestyle behaviors when children are developing their health-care habits.	Sbruzzi et, al. 2013

Author & Year	Intervention	Outcome	Results Mean Difference IV [95% CI] ([a] Hedges'g)	Conclusion of the author	Author & Year of SR
Harrison et, al. 2011	Cook lesson<br>Permitted food during breaks<br>HE policy<br>Sports during breaks<br>"Park and stride" scheme<br>PA policy<br>PA and HE policy<br>Nutrition policy	FMI	NR<br>NR<br>NR<br>NR<br>NR<br>NR<br>NR<br>NR	These data suggest that few school factors are associated with FMI, and provide limited pointers to inform potential future school-based interventions to reduce obesity.	Williams et. Al, 2013
Hoelscher et, al. 2010	Nutrition education and physical activity	Positive behavioral changes (in BPC group)<br>↓ BMI (kg/m^2) (in BPC group)	NR<br>NR	Results from this study indicate the need for community-level emphasis and involvement in school-based interventions that target underserved populations, and supports previous child obesity prevention work with CATCH	Andrade et, al. 2018
Hollar et, al. 2010	Modified cafeteria menu, nutrition/lifestyle curriculum and physical activity	↓ BMI (kg/m^2)<br>↑ BP (in males)	NR<br>NR	Holistic school-based obesity prevention interventions can improve health outcomes and academic performance, in particular among high-risk populations.	Andrade et, al. 2018
Johnson et, al. 2012	Be Active Eat Well<br>HE policy<br>PA policy	BMI (kg/m^2)<br>Adjusted change in BMI-SDS<br>Adjusted change in BMI-SDS<br>Adjusted change in BMI-SDS	0.20 [-0.08, 0.48]<br>-0.085 [-0.18,0.01] [a]<br>-0.008 [-0.06,0.04] [a]<br>-0.006 [-0.06,0.05] [a]	Obesity prevention strategies should not only target individual behaviours but also the household environment and family practices.	Williams et, al. 2013
Jordan et, al. 2008	Utah's Gold Medal Schools	Change in BMI-SDS	NR	Gold Medal Schools positively impacted body mass index z scores and health behaviors among elementary-aged students.	Williams et, al. 2013
Levy et, al. 2012	Nutrition education and physical activity	↓ BMI<br>Waist circumference	NR<br>NR		Andrade et, al. 2018

Author & Year	Intervention	Outcome	Results Mean Difference IV [95% CI] ([a] Hedges'g)	Conclusion of the author	Author & Year of SR
		BP	NR	The intervention strategy is effective in maintaining the BMI of school children.	
		Cholesterol	NR		
		Triglycerides	NR		
		No change in dietary intake	NR		
		Knowledge	NR		
Luepker et, al. 1996	Multi-component school-based intervention (CATCH program: health behavior interventions, classroom curriculum, home programs) vs. Usual curriculum, PA education and food service	BMI (kg/m^2)	0.00 [-0.27, 0.27]	The CATCH intervention was able to modify the fat content of school lunches, increase moderate-to-vigorous physical activity in PE, and improve eating and physical activity behaviors in children during 3 school years.	Sbruzzi et,al. 2013
		Systolic BP (mmHg)	0.20 [-0.35, 0.75]		
		Diastolic BP (mmHg	0.40 [-0.04, 0.84]		
Manios et, al. 2002	Multi-component workbooks vs. Unclear	BMI (kg/m^2)	-0.60 [-1.23, 0.03]	Combining the health education programme with the PE classes optimises both the physical activity and fitness as well as the nutritional and health-promotion components of the programme.	Sbruzzi et,al. 2013
Robinson 1999	Classroom curriculum to reduce screen time vs. Only assessment	Waist circumference	-1.10 [-3.43, 1.23]	Reducing television, videotape, and video game use may be a promising, population-based approach to prevent childhood obesity.	Sbruzzi et,al. 2013
		BMI (kg/m^2)	-0.10 [-1.09, 0.89]		
Safdie et, al.	Nutrition education and physical activity	Consumption of "recommended foods"	NR	The intervention improved the school food environment and child healthy behaviors.	Andrade et, al. 2018
		↓ Consumption of "sometimes foods"	NR		
		↓ Consumption of "no foods"	NR		
		↓ BMI (kg/m^2)	NR		
Siegrist et, al. 2011	Multi-component JuvenTM intervention vs. Usual activities	Waist circumference	-0.40 [-1.66, 0.86]	Children who had media consumption for more than 2 h/day did, however, report a decrease in media use.	Sbruzzi et,al. 2013
		BMI (kg/m^2)	0.20 [-0.28, 0.68]		
		BMI z-score	0.12 [-0.04, 0.28]		

Author & Year	Intervention	Outcome	Results Mean Difference IV [95% CI] ([a] Hedges'g)	Conclusion of the author	Author & Year of SR
Veugelers & Fitzgerald 2005	Nutrition policy	Adjusted odds ratio overweight	0.91 [0.77,1.09] [a]	School programs are effective in preventing childhood obesity supports the need for broader implementation of successful programs, which will reduce childhood obesity and, in the longer term, comor- bid conditions and health care spending.	Williams et, al. 2013
		Adjusted odds ratio obesity	0.85 [0.63,1.55] [a]		
	Annapolis Valley Health Promoting Schools Project	Adjusted odds ratio overweight	0.41 [0.32,0.53] [a]*		
		Adjusted odds ratio obesity	0.28 [0.14,0.57] [a]*		
Williamson et, al. 2012	Multicomponent intervention vs. No intervention	BMI z-score	-0.13 [-0.32, 0.06]	Addition of a classroom/internet program to the environmental program did not enhance weight/fat gain prevention, but did impact physical activity and social support in overweight children.	Sbruzzi et,al. 2013
	Professionally led PE	Adjusted change in BMI Healthy Fitness Zone achievement rate	0.62 [0.01,1.23] [a]*		
	Duration of PE		0.05 [-0.03,0.13] [a]		
	Number of PE periods		1.06 [0.47,1.65] [a]*		
	Duration of breaks		2.71 [1.75,3.67] [a]*		
	Number of breaks		-2.25 [-3.86, -0.64] [a]*		
	Cancelled due to weather		-1.26 [-3.73,1.21] [a]		
	PE exemptions		-0.34 [-0.65, -0.03] [a]*		
	United States Department of Agriculture wellness program		0.02 [-1.49,1.53] [a]		
	Wellness council		0.41 [-0.04,0.86] [a]		
**For Treatment**					
Aragona et, al. 1975	Parent-only reinforcement (nutritional information, exercise instruction, weight and calorie information, behavioral techniques) vs. Waiting list	Change in BMI (post intervention)	-2.28 [-3.35, -1.21] *	The response-cost group just missed significance. A 31-week, no-contact follow-up failed to show a treatment effect, but did show a trend towards slower weight gain by the response-cost plus reinforcement group.	Loveman et, al. 2015
		Change in BMI (on longest follow-up)	-1.86 [-7.39, 2.67]		
	Parent-only (nutritional information, exercise instruction, weight and calorie information) vs. Waiting list	Change in BMI (post intervention)	-2.07 [-3.81, -0.33] *		
		Change in BMI (on longest follow-up)	-0.98 [-6.95, 4.99]		

Author & Year	Intervention	Outcome	Results Mean Difference IV [95% CI] ([a] Hedges'g)	Conclusion of the author	Author & Year of SR
	Parent-only reinforcement vs. Parent-only intervention	Change in BMI (post intervention)	-0.21 [-1.59, 1.17]		
		Change in BMI (on longest follow-up)	-0.88 [-3.32, 1.56]		
Arauz Boudreau et, al. 2013	Lifestyle intervention and behavioral coaching vs. Waiting-list control	Change in BMI z-score	0.02 [-0.07, 0.11]	Latino families are willing to participate in group classes and health coaching to control childhood obesity. It may be necessary for primary care to partner with community initiatives to address childhood obesity in a more intense manner.	Mead et,al. 2017
Boutelle et, al. 2011	Dietary modifications increased physical activity, sedentary activity, behavioral change skills and parenting skills. Parent-only vs. family interventions	Change in BMI z-score (baseline to completion of treatment)	-0.02 [-0.48, 0.44]	PO treatment could provide similar results to PC in child weight loss and other relevant outcomes, and potentially could be more cost-effective and easier to disseminate.	Jull et, al. 2013 Loveman et, al. 2015
		Change in BMI z-score (baseline to completion of follow-up)	0.03 [-0.09, 0.15]		
		Change in BMI z-score (post intervention)	-0.06 [-0.18, 0.3]		
		Change in BMI z-score (on longest follow-up)	-0.02 [-0.29, 0.25]		
Bryant et, al. 2011	WATCH IT intervention vs. Waiting-list control	Change in BMI z-score	0.06 [-0.05, 0.17]	This study provided us with confidence that we can run a phase III multi-centre trial to test the effectiveness of WATCH IT. Importantly, it was invaluable in informing the design not only of that trial but also of future evaluations of childhood obesity treatment interventions.	Mead et, al. 2017
Collins et, al. 2011	Parent only diet intervention vs. Parent-child PA	Change in BMI z-score (post intervention)	-0.15 [-0.26, -0.04] *	A reduction in BMI z score was sustained at 24 months by treatment	Loveman et, al. 2015

Author & Year	Intervention	Outcome	Results Mean Difference IV [95% CI] ([a] Hedges'g)	Conclusion of the author	Author & Year of SR
	Parent only diet intervention vs. Parent-child PA + diet	Change in BMI z-score (post intervention)	0 [-0.11, 0.11]	with either program combination. The greatest effects were achieved through inclusion of a parent-centered diet program, indicating the importance of targeting parents within treatment and the possibility of targeting them exclusively in treating obese prepubertal children.	
	Parent only diet intervention vs. Parent-child PA	Change in BMI z-score (on longest follow-up)	-0.16 [-0.36, 0.04]		
	Parent only diet intervention vs. Parent-child PA + diet	Change in BMI z-score (on longest follow-up)	-0.11 [-0.31, 0.09]		
Coppins et, al. 2011	Family-focused education package vs. Waiting-list control	Change in BMI z-score	0.01 [-0.17, 0.19]	Children given active intervention followed by body composition monitoring alone reduced their BMI SDS, and fewer children were classified as grossly overweight by the end of the study. If these findings are true, there are important implications for the provision of services managing overweight in the community.	Mead et, al. 2017
		Change in Weight (kg)	-1.20 [-4.20, 1.80]		
Croker et, al. 2011	Advice on whole-family lifestyle change, behavioral weight control program vs. Waiting list control	Waist circumference	-0.90 [-2.43, 0.63]	Both treatment and control groups experienced significant reductions in the level of overweight, but with no significant difference between them. There were no significant group differences for any of the secondary outcomes.	Sbruzzi et,al. 2013
		BMI (kg/m^2)	-0.50 [-1.09, 0.09]		
		BMI z-score	0.02 [-0.06, 0.10]		
Croker et, al. 2012	Family-based behavioral treatment vs. Waiting list control	Change in BMI (kg/m^2)	-0.33 [-0.86, 0.20]	Both treatment and control groups experienced significant reductions in level of overweight, but with no significant difference between them. There were no significant group differences for any of the secondary outcomes.	Mead et, al. 2017
		Change in BMI z-score	-0.01 [-0.09, 0.07]		
		Change in Weight (kg)	-1.99 [-3.40, -0.58]		

Author & Year	Intervention	Outcome	Results Mean Difference IV [95% CI] ([a] Hedges'g)	Conclusion of the author	Author & Year of SR
Davis et, al. 2013	Telemedicine intervention vs. Physician- visit	Change in BMI z-score	0.03 [-0.29, 0.35]	Both telemedicine and structured physician visit may be feasible and acceptable methods of delivering pediatric obesity treatment to rural children.	Mead et, al. 2017
Davoli et, al. 2013	Family-pediatrician led motivational interviewing (multidisciplinary behavioral group) vs. Usual care + booklet on obesity prevention	Change in BMI (kg/m^2)<br>Change in BMI z-score	-0.04 [-0.36, 0.28]<br>-0.02 [-0.11, 0.07]	The pediatrician-led MI was overall effective in controlling BMI in these overweight children aged 4 to 7 years, even though no effect was observed in male children or when the mother's education level was low.	Mead et, al. 2017
Diaz et, al. 2010	Behavioral curriculum + dietitian and physician consultation vs. Physician consultation only	Change in BMI (kg/m^2)<br>Change in BMI z-score<br>Change in Weight (kg)	-1.20 [-2.22, -0.18] *<br>-0.20 [-0.34, -0.06] *<br>-3.50 [-6.40, -0.60] *	Lifestyle intervention in primary care settings improves obesity parameters among Mexican youth.	Mead et, al. 2017
Eddy Ives et, al. 2012	Diet and PA recommendation (6 sessions) vs. Waiting list control (2 sessions)	Change in BMI (kg/m^2)<br>Change in BMI z-score<br>Change in Weight (kg)	0.07 [-0.93, 1.07]<br>0.04 [-0.16, 0.24]<br>-0.15 [-3.81, 3.51]	Lifestyle counseling was effective to reduce body mass index and waist circumference in adolescents, regardless of intensity. Counseling improved food habits, but not exercise.	Mead et, al. 2017
Epstein 2000	Behavioral wight-control program + parent-child problem solving vs. Behavioral wight-control program + child-only problem solving	Change in BMI z-score<br>Change in Weight (kg)	0.39 [-0.15, 0.93]<br>1.78 [-5.23, 8.78]	Parents and children can lose significant amounts of weight over a 1-year period.	Mead et, al. 2017
Esfarjani et, al. 2013	Training program 12 sessions (strategies development,	Change in BMI (kg/m^2) (post intervention)	-0.4 [-1.18, 0.38]	The family-based lifestyle program had limited but desirable effects on	Loveman et, al. 2015

Author & Year	Intervention	Outcome	Results Mean Difference IV [95% CI] ([a] Hedges'g)	Conclusion of the author	Author & Year of SR
	nutrition, exercise habits and guidelines, reduce sedentary behaviors) vs. 2 sessions (no details provided)	Change in BMI (kg/m^2) (on longest follow-up)	-0.5 [-1.3, 0.3]	anthropometric and metabolic outcomes of the obese children. We suggest that a longer period of intervention may have more favorable results.	
Gillis et, al. 2007	Exercise + diet education with weekly diaries and calls vs. Exercise +diet education only	Change in BMI z-score	-0.12 [-0.25, 0.01]	Targeting cheaper effective therapy is imperative in order to curb the ongoing obesity epidemic which is now attacking poorer populations. Our study is encouraging as it shows a clear trend in the direction of success.	Mead et,al. 2017
Golan et, al. 2006	Healthy eating, increased physical activity and decreased physical activity. Parent-only vs. family intervention.	z-BMI (baseline to completion of treatment)	-0.30 [-0.91, 0.31]	Targeting the health-centred approach to childhood obesity with the parents as the exclusive mediator, resulted in better results than the situation where parents attended sessions with the obese child.	Jull et, al. 2013 Mead et, al. 2017
Golley et, al. 2007	Parenting-skills training + intensive lifestyle education vs. Parenting-skills training vs. Waiting list control	BMI z-score	-0.08 [-0.33, 0.17]	Parenting-skills training combined with promoting a healthy family lifestyle may be an effective approach to weight management in prepubertal children, particularly boys. Future studies should be powered to allow gender subanalysis.	Sbruzzi et,al. 2013 Loveman et, al. 2015
	Parenting-skills training vs. Waiting list control	Change in BMI z-score (on longest follow-up)	-0.11 [-0.36, 0.14]		
	Parenting-skills training + intensive lifestyle education vs. Waiting list control	Change in BMI z-score (on longest follow-up)	-0.02 [-0.29, 0.25]		
	Parenting-skills training + intensive lifestyle education vs. Parenting-skills training	Change in BMI z-score (post intervention)	-0.09 [-0.38, 0.2]		
		Change in BMI z-score (on longest follow-up)	-0.09 [-0.32, 0.14]		
Gunnarsdottir et, al. 2011a	Epstein's family-based behavioural treatment (FBBT) vs. Waiting list control	Change in BMI z-score	-0.37 [-0.84, 0.11]	Treatment was found acceptable and effects were promising. These results	Mead et,al. 2017

Author & Year	Intervention	Outcome	Results Mean Difference IV [95% CI] ([a] Hedges'g)	Conclusion of the author	Author & Year of SR
				provide substantiation for a larger study of treatment effects.	
Janicke et, al. 2008	Healthy dietary habits (modified traffic light diet), increased physical activity (podometer use, decrease sedentary activity), build self-worth vs. waiting-list	Change in BMI z-score (baseline to completion of treatment)	-0.22 [-0.64, 0.20]	A parent-only intervention may be a viable and effective alternative to family-based treatment of childhood overweight. Cooperative Extension Service offices have the potential to serve as effective venues for the dissemination of obesity-related health pro- motion programs.	Jull et, al. 2013 Loveman et, al. 2015
		Change in BMI z-score (baseline to completion of follow-up)	0.03 [-0.09, 0.15]		
	Parent-only (behavioral, nutritional and PA) vs. Parent-child (behavioral, nutritional and PA)	Change in BMI z-score (post intervention)	-0.06 [-0.15, 0.03]		
		Change in BMI z-score (on longest follow-up)	0.06 [-0.05, 0.16]		
	Parent-only (behavioral, nutritional and PA) vs. Waiting list control	Change in BMI z-score (post intervention)	-0.13 [-0.22, -0.04] *		
		Change in BMI z-score (on longest follow-up)	-0.11 [-0.22, -0]		
Kalarchian et, al. 2009	Multi-component family-based intervention (behavioral strategies for PA and sedentary behaviors) vs. Usual care	Change in BMI (kg/m^2)	-0.22 [-0.94, 0.50]	Intervention was associated with significant short-term reductions in obesity and improvements in medical parameters and conferred longer-term weight change benefits for children who attended > or =75% of sessions.	Mead et, al. 2017 Sbruzzi et,al. 2013
		BMI (kg/m^2)	-2.10 [-3.49, -0.71] *		
		Waist circumference	-6.10 [-11.07, -1.13] *		
		Systolic BP (mmHg)	-5.80 [-8.53, -3.07] *		
		Diastolic BP (mmHg	-3.60 [-5.91, -1.29] *		
Kalavainen et, al. 2007	Multi-component family-based intervention (nutritional education, PA and behavioral therapy) vs. Routine counselling (2 appointments for children)	BMI (kg/m^2)	-0.30 [-1.45, 0.85]	Family-based group treatment that stresses a health-promoting lifestyle and is given separately for parents and children, offers an effective mode of therapy to treat obese school-aged children.	Mead et, al. 2017 Sbruzzi et,al. 2013
		Change in BMI (kg/m^2)	-0.20 [-1.31, 0.91]		
		Change in BMI z-score	0.00 [-0.24, 0.24]		
		BMI z-score	0.00 [-0.28, 0.28]		

Author & Year	Intervention	Outcome	Results Mean Difference IV [95% CI] ([a] Hedges'g)	Conclusion of the author	Author & Year of SR
Kirk et, al. 2012	Low carbohydrate diet + group exercise/education sessions vs. Reduced glycaemic load diet + group exercise/education sessions vs. Standard portion-controlled diet + group exercise/education sessions	Change in BMI z-score	0.07 [-0.08, 0.22]	Diets with modified CHO intake were as effective as a PC diet for weight management in obese children. The lower adherence to the LC diet suggests that this regimen is more difficult for children to follow, particularly in the long term.	Mead et, al. 2017
Lison et, al. 2012	Hospital clinic group exercise-diet program vs. Home-based exercise-diet program vs. Usual care	Change in BMI (kg/m^2)<br>Change in BMI z-score	-2.40 [-4.31, -0.49] *<br>-0.18 [-0.31, -0.06] *	Simple home-based combined exercise and Mediterranean diet program may be effective among overweight and obese children and adolescents, because it improves body composition, is feasible and can be adopted on a large scale without substantial expenses.	Mead et, al. 2017
Looney et, al. 2014	Newsletter and growth monitoring + behavioral counselling vs. Newsletter and growth monitoring vs. Newsletter only	Change in BMI z-score	-0.05 [-0.52, 0.42]	Low-intensity obesity treatments can reduce z-BMI and may be more feasible in primary care.	Mead et, al. 2017
Maddison et, al. 2014	SWITCH intervention vs. usual care	Change in BMI (kg/m^2)<br>Change in BMI z-score<br>Change in Weight (kg)	-0.01 [-1.29, 1.27]<br>-0.03 [-0.26, 0.20]<br>0.21 [-429, 4.71]	A home-based, family-delivered intervention to reduce all leisure-time screen use had no significant effect on screen-time or on BMI at 24 weeks in overweight and obese children aged 9-12 years.	Mead et, al. 2017
Magarey et, al. 2011	Parenting healthy lifestyle (Positive Parenting Program) vs. Healthy lifestyle (recommendations on nutrition, PA, sedentary behaviors)	Change in BMI z-score (post intervention)<br>Change in BMI z-score (on longest follow-up)	-0.07 [-0.29, 0.15]<br>0.03 [-0.24, 0.3]	Using approaches that specifically target parent behavior, relative weight loss of ~10% is achievable in moderately obese prepubertal children and can be maintained for 2 years from baseline. These results justify an investment in	Loveman et, al. 2015

Author & Year	Intervention	Outcome	Results Mean Difference IV [95% CI] ([a] Hedges'g)	Conclusion of the author	Author & Year of SR
				treatment as an effective secondary obesity-prevention strategy.	
Markert et, al. 2014	Family telephone-based adiposity prevention vs. No care	Change in BMI z-score	-0.03 [-0.12, 0.06]	A telephone-based obesity prevention program suffers from well-known high attrition rates so that its effectiveness could only be shown in those who adhered to completion.	Mead et, al. 2017
Mazzeo et, al. 2014	NOURISH intervention (parent-only skills-based intervention) vs. Control (group session)	Change in BMI percentile (post intervention)	-0.28 [-1.38, 0.82]	NOURISH is acceptable and, with refinement, offers promise for reducing pediatric BMI. Outcomes, lessons learned, and parent feedback will inform a larger randomized controlled trial.	Loveman et, al. 2015
McCallum et, al. 2007	LEAP intervention vs.No care	Change in BMI (kg/m^2)<br>Change in BMI z-score	0.00 [-0.81, 0.81]<br>-0.02 [-0.21, 0.17]	Brief individualized solution-focused approaches may not be an effective approach to childhood overweight.	Mead et, al. 2017
Mirza et, al. 2013	Low-glycaemic load diet vs. Conventional low-fat diet	Change in BMI z-score	-0.07 [-0.17, 0.03]	LGD and an LFD differ in efficacy for the reduction of BMI or aspects of metabolic syndrome in obese Hispanic youth. Both diets decreased the BMI z score when prescribed in the context of a culturally adapted, comprehensive weight-reduction program.	Mead et, al. 2017
Nemet et, al. 2005	Dietary and exercise program vs. Usual care	Change in BMI (kg/m^2)<br>Change in Weight (kg)	-2.20 [-5.26, 0.86]<br>-4.60 [-17.49, 8.29]	The short- and longer-term beneficial effects of a combined dietary-behavioral-physical activity intervention among obese children.	Mead et, al. 2017
O'Connor et, al. 2013	Helping HAND obesity intervention vs. Waiting list control	Change in BMI z-score	0.05 [-0.16, 0.26]	Helping HAND is feasible, due to low attrition, good programme attendance, and clinically relevant improvements in some child and parenting behaviours.	Mead et, al. 2017

Author & Year	Intervention	Outcome	Results Mean Difference IV [95% CI] ([a] Hedges'g)	Conclusion of the author	Author & Year of SR
Raynor 2012a	Behavioral intervention (parent-only vs. decreased intake)	Change in BMI z-score (post intervention)	-0.04 [-0.17, 0.09]	All interventions improved weight status.	Loveman et, al. 2015
		Change in BMI z-score (on longest follow-up)	-0.04 [-0.19, 0.11]		
	Behavioral intervention (parent-only vs. increased intake)	Change in BMI z-score (post intervention)	-0.01 [-0.14, 0.12]		
		Change in BMI z-score (on longest follow-up)	-0.02 [-0.17, 0.13]		
Raynor 2012	Behavioral intervention (parent-only vs. substitute intervention)	Change in BMI z-score (post intervention)	-0.7 [-0.86, -0.54] *		Loveman et, al. 2015
		Change in BMI z-score (on longest follow-up)	-0.03 [-0.24, 0.18]		
	Behavioral intervention (parent-only vs. traditional intervention)	Change in BMI z-score (post intervention)	-0.69 [-0.83, -0.55] *		
		Change in BMI z-score (on longest follow-up)	0.01 [-0.17, 0.19]		
Resnick et, al. 2009	Educational material + personal encounter (biological, social and environmental influences; nutrition advice and PA guidelines) vs. Educational material (increased PA and nutritional components)	Change in BMI z-score (post intervention)	1.2 [-3.89, 6.29]	School nurses, working in concert with an academic team, can successfully organize a team of community workers while retaining a high proportion of parents 1 year after the onset of study recruitment.	Loveman et, al. 2015
		Change in BMI percentile (post intervention)	1.1 [-1.59, 3.79]		
Resnicow et, al. 2015	MI (counseling, diet education, healthy lifestyle, positive feedback, self-monitoring) vs. Waiting list-control	Change in BMI percentile (post intervention)	-2 [-5.3, 1.3]	MI delivered by providers and RDs (group 3) resulted in statistically significant reductions in BMI percentile.	Loveman et, al. 2015
	MI (counseling, diet education, healthy lifestyle, positive feedback, self-monitoring) + dietician vs. Waiting list-control	Change in BMI percentile (post intervention)	-3.1 [-6.42, 0.22]		

Author & Year	Intervention	Outcome	Results Mean Difference IV [95% CI] ([a] Hedges'g)	Conclusion of the author	Author & Year of SR
Rodearmel et, al. 2007	America on the Move intervention vs. Self-monitoring	Change in BMI z-score	-0.03 [-0.08, 0.02]	The small-changes approach advocated by America on the Move could be useful for addressing childhood obesity by preventing excess weight gain in families.	Mead et, al. 2017
Sacher et, al. 2010	Multi-component family-based intervention (MEND program) vs. 6 month-delayed intervention	BMI (kg/m^2)	-2.00 [-3.60, -0.40]	High-attendance rates suggest that families found this intensive community-based intervention acceptable.	Sbruzzi et,al. 2013<br>Mead et, al. 2017
		Change in BMI (kg/m^2)	-2.10 [-3.96, -0.24] *		
		Change in BMI z-score	-0.29 [-0.54, -0.04] *		
		BMI z-score	-0.28 [-0.49, -0.07] *		
		Waist circumference	-4.30 [-7.20, -1.40] *		
		Systolic BP (mmHg)	-1.40 [-4.90, 2.10]		
		Diastolic BP (mmHg	-3.80 [-6.66, -0.94] *		
Saelens et, al. 2013	Self-directed intervention vs. Prescribed approach	Change in BMI z-score	-0.07 [-0.27, 0.13]	An adjunct motivational and autonomy-enhancing approach to behavioral family-based pediatric obesity treatment is a viable alternative to the standard intervention approach.	Mead et, al. 2017
Serra-Paya et, al. 2015	Nereu program vs. Counselling group	Change in BMI z-score	-0.3 [-0.11, 0.05]	8-month intervention of the Nereu Program group showed improvement in physical activity and dietary behaviours, compared to the counselling group.	Mead et, al. 2017
Siwik et, al. 2013	'Choices' group office-visit intervention vs. control group	Change in BMI (kg/m^2)	-0.40 [-3.95, 3.15]	The innovative approach used in this study demonstrated modest efficacy in reducing BMI z score, changing physical activity levels, and possibly shifting family dynamics.	Mead et, al. 2017
		Change in BMI z-score	-0.04 [-0.34, 0.26]		
		Change in Weight (kg)	-0.50 [-9.28, 8.29]		

Author & Year	Intervention	Outcome	Results Mean Difference IV [95% CI] ([a] Hedges'g)	Conclusion of the author	Author & Year of SR
Small et, al. 2013	Educational intervention (healthy habits, nutrition, PA, sedentary behaviors) and MI with follow-up vs. Educational information on age-appropriate health and safety	Change in BMI percentile (post intervention)<br>Change in BMI percentile (on longest follow-up)	-2.32 [-5.55, 0.91]<br>-0.93 [-3.49, 1.63]	Primary care-based, parent-focused overweight/obesity treatment program is feasible and demonstrated positive preliminary effects, improving the children's overall health trajectory.	Loveman et, al. 2015
Taylor et, al. 2015	Tailored package family-based interventions vs. Usual care	Change in BMI (kg/m^2)<br>Change in BMI z-score<br>Change in Weight (kg)	-0.40 [-1.18, 0.38]<br>-0.15 [-0.29, -0.01] *<br>-0.60 [-3.31, 2.11]	Low-dose support was effective for reducing excessive weight in predominantly mild to moderately overweight children over a 2-year period. Such initiatives could feasibly be incorporated into primary care.	Mead et, al. 2017
Van Grieken et, al. 2013	MI and information on healthy lifestyle choices vs. Usual care	Change in BMI (kg/m^2) (in longest follow-up)	-0.07 [-0.36, 0.22]	The intervention condition showed a significantly smaller increase in BMI at follow-up compared to the control condition; there was no overall difference between intervention and control condition.	Loveman et, al. 2015
Wafa et, al. 2011	Multi-component family-based intervention (sedentary behavior, PA and diet with behavior change counseling) vs. 6 moths-delayed intervention	Change in BMI z-score<br>BMI z-score<br>Change in Weight (kg)	-0.10 [-0.38, 0.18]<br>-0.15 [-0.39, 0.09]<br>-2.00 [-3.02, -0.98]	Treatment was associated with reduced rate of weight gain, and improvements in physical activity and quality of life.	Mead et,al. 2017<br>Sbruzzi et,al. 2013
Wake et, al. 2009	LEAP2 behavior intervention vs. No care	Change in BMI (kg/m^2)	-0.10 [-0.70, 0.50]	Primary care screening followed by brief counselling did not improve BMI, physical activity, or nutrition in overweight or mildly obese 5-10 year olds, and it would be very costly if universally implemented.	Mead et,al. 2017
Wake et, al. 2013	HopSCOTCH (the shared care obesity trial) intervention vs. Usual care	Change in BMI (kg/m^2)<br>Change in BMI z-score	0.10 [-1.37, 1.57]<br>-0.10 [-0.27, 0.07]	The shared care model of primary and tertiary care management did not lead to better body mass index or other	Mead et,al. 2017

Author & Year	Intervention	Outcome	Results Mean Difference IV [95% CI] ([a] Hedges'g)	Conclusion of the author	Author & Year of SR
				outcomes for the intervention group compared with the control group. Improvements in body mass index in both groups highlight the value of untreated controls when determining efficacy.	
Wailing et, al. 2012	Family-based intervention vs. Usual care	Change in BMI (kg/m^2)<br>Change in BMI z-score<br>Change in Weight (kg)	-0.30 [-1.58, 0.98]<br>0.01 [-0.16, 0.18]<br>-2.10 [-7.29, .09]	The intervention had limited effects on anthropometrics and metabolic markers, which emphasizes the need of preventing childhood overweight and obesity.	Mead et,al. 2017
Warschburger et, al. 2016	Parental CBT training group + child inpatient intervention vs. Parental information-only group plus child inpatient intervention	Change in BMI z-score	0.02 [-0.06, 0.10]	While the inpatient treatment proved highly effective, additional parent training did not lead to better results in long-term weight maintenance or to better psychosocial well-being compared to written psycho-educational material.	Mead et,al. 2017
Weigel et, al. 2008	Active intervention group vs. Usual care	Change in BMI (kg/m^2)<br>Change in BMI z-score	-4.30 [-6.00, -2.60] *<br>-0.60 [-0.86, -0.34] *	Group-based programs for young, obese patients can be effective tools for establishing a health-oriented lifestyle and reducing the burden of obesity.	Mead et,al. 2017
West et, al. 2010	Parent-only behavioral change (positive-parenting, PA and nutrition strategies) vs. Waiting list control	Change in BMI z-score (post intervention)	-0.1 [-0.28, 0.08]	The results support the efficacy of Group Lifestyle Triple P and suggest that parenting influences treatment outcomes	Loveman et, al. 2015

[1] [a] PO: Parents only group. [b] Parent-child group. SWITCH: Screen-Time Weight-loss Intervention Targeting Children at Home; MI: Motivational interviewing; PA: Physical activity; IVR: Interactive Voice Response; SD: Standard Deviation; CI: Confidence Interval; BMI: Body Mass Index; OR: Odds Ratio; LNED: Low-nutrient, energy-dense ; PC: Portion controlled; LC: Low carbohydrate; LLD: Low glycemic-load diet; LFD: Low fat diet. NR: No Report.
